# Genetic profiling links changing sea-ice to shifting beluga whale migration patterns

**DOI:** 10.1098/rsbl.2016.0404

**Published:** 2016-11

**Authors:** Greg O'Corry-Crowe, Andrew R. Mahoney, Robert Suydam, Lori Quakenbush, Alex Whiting, Lloyd Lowry, Lois Harwood

**Affiliations:** 1 Harbor Branch Oceanographic Institute, Florida Atlantic University, Fort Pierce, FL 34946, USA; 2 Geophysical Institute, University of Alaska, Fairbanks, AK 99708, USA; 3 Department of Wildlife Management, North Slope Borough, Barrow, AK 99723, USA; 4 Alaska Department of Fish and Game, Fairbanks, AK 99701, USA; 5 Native Village of Kotzebue, Kotzebue, AK 99752, USA; 6 Alaska Beluga Whale Committee, Barrow, AK 99723, USA; 7 Department of Fisheries and Oceans, Yellowknife, Northwest Territories, Canada X1A 1E2

**Keywords:** migration, sea-ice, climate change, beluga whale, *Delphinapterus leucas*, killer whale

## Abstract

There is increasing concern over how Arctic fauna will adapt to climate related changes in sea-ice. We used long-term sighting and genetic data on beluga whales (*Delphinapterus leucas*) in conjunction with multi-decadal patterns of sea-ice in the Pacific Arctic to investigate the influence of sea-ice on spring migration and summer residency patterns. Substantial variations in sea-ice conditions were detected across seasons, years and sub-regions, revealing ice–ocean dynamics more complex than Arctic-wide trends suggest. This variation contrasted with a highly consistent pattern of migration and residency by several populations, indicating that belugas can accommodate widely varying sea-ice conditions to perpetuate philopatry to coastal migration destinations. However, a number of anomalous migration and residency events were detected and coincided with anomalous ice years, and in one case with an increase in killer whale (*Orcinus orca*) sightings and reported predation on beluga whales. The behavioural shifts were likely driven by changing sea-ice and associated changes in resource dispersion and predation risk. Continued reductions in sea-ice may result in increased predation at key aggregation areas and shifts in beluga whale behaviour with implications for population viability, ecosystem structure and the subsistence cultures that rely on them.

## 1. Introduction

Declines in Arctic sea-ice are arguably the most dramatic evidence of the effects of current climate warming on ocean systems. How such declines reverberate through marine ecosystems is largely unknown [1]. In upper trophic level (UTL) species the first discernible effects of changing sea-ice will likely be behavioural either by directly altering ranging patterns [2,3] or indirectly by influencing resource (food, mates, breeding sites) and risk (e.g. predation) dispersion. Understanding links between changing sea-ice and shifts in behaviour of these UTL species has immediate relevance to species management as well as broader import for understanding ecosystem resilience. Here we assess the relationship between changing sea-ice and beluga whale migration and summer residency patterns of a number of populations over two decades of dramatic sea-ice changes in the Pacific Arctic.

Beluga whales predictably return to specific coastal locations each spring and summer [4]. Genetic and telemetry studies have identified several discrete populations that follow traditional migratory routes between wintering areas and summering grounds where belugas feed, moult and raise their young (e.g. [5,6]). It is not clear how sea-ice influences these migrations and summer habitat use. Climate change has added urgency to determining how environmental factors might shape the behaviour and ecology of this species.

We used genetic data to investigate the population of origin of whales returning to four traditional coastal sites in the Alaskan and Canadian Arctic between 1988 and 2007. We compiled detailed beluga sighting and harvest data for the same period to assess inter-annual variation in timing of return. Finally, we analysed sea-ice data in the Bering, Chukchi and Beaufort seas to determine seasonal and regional patterns of sea-ice from 1979 to 2014. We addressed two specific questions: (i) how does the pattern of annual return to a particular coastal area correlate with inter-annual variation in sea-ice and (ii) do anomalies in beluga migration behaviour correlate with sea-ice anomalies?

## 2. Material and methods

Tissue samples from 978 beluga whales were collected over a 30-year period from subsistence harvests and from live whales via biopsy. Total DNA was extracted from each sample and screened for variation within 410 bp of the mitochondrial genome and for polymorphism within eight hypervariable microsatellite markers, according to previously published methods [5]. Population structure and population of origin of individuals were assessed using: (i) homogeneity tests of genetic differentiation in Arlequin (V3.5) [7] and (ii) clustering analysis and assignment tests using Structure (V2.3.4) [8] and Whichrun (V4.1) [9].

**Figure 1.**
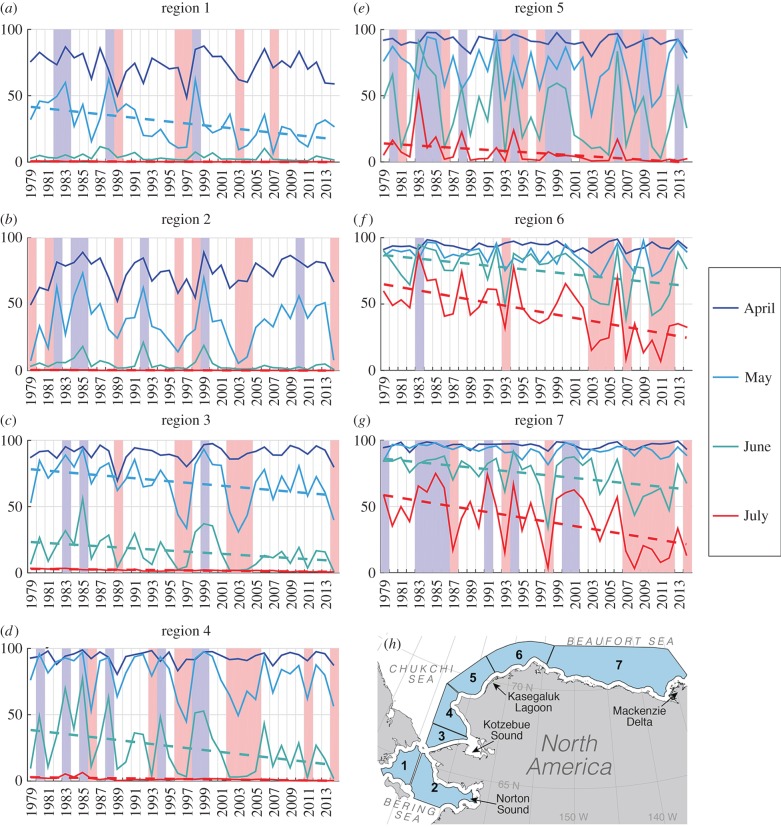
Regional (*n* = 7) sea-ice concentration (%) in the Bering, Chukchi and Beaufort seas from 1979 to 2014. Positive ice anomaly years are highlighted as blue bars, negative anomaly years as pink bars. Trends are indicated by dashed lines.

Minimum estimates of the period of annual return by beluga whales to Kasegaluk Lagoon and the Mackenzie Delta (figure 1*h*) were based on sightings of whales by native hunters and field biologists, from aerial surveys and from the timing of subsistence harvests ([10,11]; electronic supplementary material).

Variability in sea-ice conditions was examined using passive microwave-derived sea-ice concentration (SIC) data [12] for the years 1979–2014. Sea-ice conditions on spatial scales of relevance to the migrations of different beluga populations were assessed by calculating monthly mean SICs for seven regions within our study area (figure 1), excluding waters within 25 km of the coast where data may be biased by proximity to land. To identify years with anomalous ice conditions, months in which beluga typically occupy each region (April–May for regions 1–3; May–June for regions 4, 5 and June–July for regions 6, 7) were examined and anomalous occasions when the monthly SIC was 20‰ above or below the 36-year mean were identified.

## 3. Results

**Figure 2.**
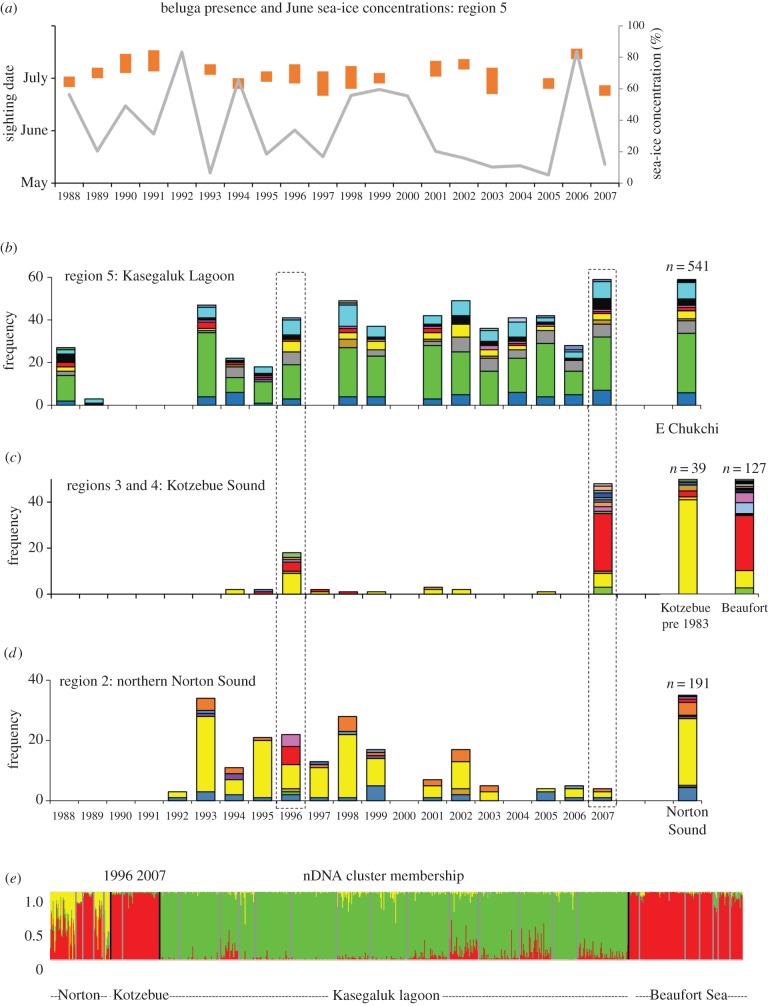
The timing of occurrence and population of origin of beluga whales from a number of locations in the Alaskan Arctic. (*a*) Beluga presence (orange bars) and sea-ice trends (grey line) for sea-ice region 5. Frequency of mtDNA lineages (coloured bars) from whales sampled in (*b*) Kasegaluk Lagoon, (*c*) Kotzebue Sound and (*d*) Norton Sound for the period 1988–2007, compared with total population frequencies. (*e*) Population cluster membership (coloured bars) of 816 individual whales based on eight microsatellite loci. Individuals dominated (i.e. *Q* > 0.8) by the same colour profile are considered part of the same population. Black lines demarcate the different locations and grey lines demarcate the different sample years. Only years with more than five samples were included.

Beluga whales occur in the eastern Chukchi Sea (sea-ice region 5, figure 1*h*) near shore waters each summer. For the years 1988–2007, beluga presence based on sightings ranged from as early as 20 June (1997) to as late as 16 July (1991, 2006) (figure 2*a*). Mean June SIC for this region varied considerably from 5.2% (1997) to 83.7% (2006) and was weakly correlated with the timing of whale sightings near shore (*ρ* = 0.36, figure 2*a*). A consistent genetic profile was found for both mtDNA (figure 2*b*) and nDNA (figure 2*e*) for beluga whales occurring off Kasegaluk Lagoon in June and July, indicating that the same, distinct population returned at roughly the same time each year over the course of the study despite high inter-annual variation in sea-ice. Similar patterns were found for the Mackenzie Delta (figure 2*e*; electronic supplementary material, tables SM1 to SM4).

Prior to 1983, beluga whales returned to Kotzebue Sound in the southeastern Chukchi Sea (map, figure 1*h*) each summer with a peak in mid-June. The genetic signature across years denoted a distinct Kotzebue population (figure 2*c*). After 1983, the occurrence of whales in Kotzebue Sound decreased dramatically. Two exceptions to this occurred in 1996 and 2007 when several hundred whales entered the sound. The genetic profile of these whales was distinct from the earlier period (figure 2*c*). The 1996 event occurred within the same timeframe (10–28 June) as pre-1983 occurrences. The 2007 event primarily occurred five weeks later (23–28 July).

In Norton Sound in the eastern Bering Sea a consistent mtDNA profile was observed in most years (figure 2*d*) except for 1996 when whales from another population entered the northern part of the Sound (Norton Bay) in May. The microsatellite pattern was less distinct as Norton Sound does not form a distinct nDNA cluster (figure 2*e*).

The genetic analysis determined that all three atypical events involved whales most likely from the eastern Beaufort population which winters in the western Bering, migrates through the Chukchi and summers in the Beaufort Sea (figure 2*b*–*e*). Both sexes occurred in all three events although the Kotzebue Sound 2007 event comprised males primarily (more than 90%), differing significantly from a sex ratio of near parity for most other years sampled (*p* = 0.018).

The springtime sea-ice regime of the northern Bering, eastern Chukchi and southern Beaufort seas is characterized by a northward ice retreat from regions 1 to 7, but there is considerable variability both inter-annually and between regions (figure 1). In line with Arctic-wide trends all regions show a significant negative trend in ice concentration in July (slope = −0.1 to −11.6, *p* < 0.05) and there are more negative anomaly years in the latter half of the record.

Among the years identified as individually anomalous for each region certain years stand out as being anomalous throughout the study area. For example, 1983 was a positive anomaly in all regions except region 2, while 2003 was a negative anomaly in all regions except region 7. 1996 was a negative anomaly during April and May in regions 1–4, corresponding to an early break-up in Norton and Kotzebue sounds; whereas 2007 was a negative anomaly from May to July in regions 5–7. Ice conditions in Kasegaluk Lagoon (region 5) show especially strong inter-annual variability, with 26 out of 36 years being either positive or negative anomalies. A significant association was found between the atypical whale migration events recorded in Norton and Kotzebue sounds and negative ice anomalies in the northern Bering Sea (region 1—Norton: *r* = 0.66, *p* = 0.05, χ^2^ *p* = 0.047; region 1—Kotzebue: *r* = 0.65, *p* < 0.01, χ^2^ *p* = 0.016; region 2—Norton: *r* = 0.66, *p* = 0.05, χ^2^ *p* = 0.047).

## 4. Discussion

The relationship between Arctic whales and sea-ice is still largely a mystery. This study revealed that beluga whales are resilient to widely varying ice conditions while perpetuating philopatry each year to discrete coastal locations and suggests that reaching these areas at this time of year is critical. The detection of behavioural shifts at a number of locations in 1996 and 2007 indicates that belugas can also dramatically alter migration course and summer habitat use. That these events coincided with anomalous spring and summer sea-ice conditions is suggestive of a link between the two phenomena. That the wayward whales were up to 1800 km from their normal course indicates that the effect can be substantial. It should be noted, there were other anomalously low ice years (figure 1) when we either had few or no samples (Kasegaluk, Norton, Kotzebue) or sightings (Kotzebue) to assess possible interactions between the cryosphere and whale ranging patterns.

Sea-ice patterns in early spring (April–May) in the northern Bering Sea and in summer (June–July) in the Chukchi and Beaufort seas could be key. Early break-up should facilitate rather than impede migration of Beaufort belugas to their traditional summering area in the Mackenzie Delta, and indeed no anomalies in the period of occurrence of whales in the delta were detected for 1996 or 2007 [11], which might suggest a population-wide phenomenon. It thus, appears that some Beaufort belugas either remained in the Bering and Chukchi seas or returned westward from their summering ground in years when ice conditions were atypically low. That the Kotzebue 2007 event comprised mainly males adds to a growing body of evidence of sex segregation in belugas [13] and indicates how changes in ecosystem forcing may affect males and females differently.

Unusual sea-ice patterns may have had a more indirect effect where beluga whales altered their behaviour to pursue associated shifts in prey or to avoid changing predation risk. Recent warming is causing changes in fish communities in the Arctic [14]. As yet, there is limited information on changes in prey availability for belugas in Alaskan waters [15,16]. Native hunters have reported recent increases in killer whale sightings in Kotzebue Sound including the presence of killer whales during the 2007 event when evasive behaviour by the beluga whales and predation by killer whales was observed. This follows an Arctic-wide increase in killer whale sightings which has been linked to decreasing sea-ice [17].

It is possible that other unknown proximate factors, including anthropogenic activities, could have contributed to the altered whale migration patterns. Here we proffer an ecosystem-level, basin-wide perspective of likely ultimate and proximate causes. Our findings reveal a complex relationship between a UTL species and the Arctic cryosphere, where responses to changing sea-ice are influenced by the changing physical challenges and by biological dictates. Beluga whales exhibit great resolve in reaching critical stationary resources such as coastal moulting and breeding sites. However, changes in prey availability and predation pressure will require an adjustment in movement and habitat use patterns. Decreasing sea-ice will likely increase predation risk as temperate apex predators including killer whales have easier and longer access to the Arctic. Behavioural and ecological responses of beluga whales to all these changes may alter the pattern of return to traditional coastal sites, with possible cascading effects throughout entire marine ecosystems and the subsistence communities that rely on them.

## Supplementary Material

O'Corry-Crowe et al - Biology Letters - Supplementary Mat

## Supplementary Material

O'Corry-Crowe et al - Biology Letters - Supplementary Tables

## Data accessibility

The genetic data are available from the Dryad Digital [18] Repository; http://dx.doi:10.5061/dryad.h172b. Some of the mtDNA data were also previously submitted to GenBank: GU985185 to GU985200. The sea-ice data are available from the US National Snow and Ice Data Center website, http://nsidc.org/data/nsidc-0051.html.
