# Supplementary material for: Genetic profiling links changing sea-ice to shifting beluga whale migration patterns
Source: Biol Lett. 2016 Nov;12(11):20160404. doi: 10.1098/rsbl.2016.0404 (PMC5134032; doi:10.1098/rsbl.2016.0404)

Biology Letters

**Genetic profiling links changing sea ice to shifting beluga whale migration patterns**

by Greg O’Corry-Crowe, Andrew R. Mahoney, Robert Suydam, Lori Quakenbush, Alex Whiting, Lloyd Lowry, and Lois Harwood

Supplementary Material

**Materials and Methods**

*Genetic analysis*

Details of lab and data analysis methods are provided in earlier publications. We also provide details on the data analysis in the results section below. Some of the genetic data used in this study has been published in some form elsewhere [1,2,3,4]. The majority, however, has not been published before. Of the 977 whales included in this study, sequence data from 609 individuals and genotype data from 656 whales are presented here for the first time. All sequence and genotypic data has been submitted to GenBank and DRYAD data repositories, respectively and will also be supported on FAU’s data portal. Our DOI data package identifier on the DRYAD data repository is: doi:10.5061/dryad.h172b. We uploaded two files comprising all the genetic data; one with all the mtDNA sequence data and one with all the microsatellite genotype data. A number of mtDNA haplotypes had previously been submitted to GenBank with the following accession numbers: GU985185 to GU985200. Furthermore, we provide baseline mtDNA haplotype and microsatellite allele frequency data in Table SM1 and Table SM2.

*Beluga sighting data*

Minimum estimates of the period of annual return by beluga whales to waters off Kasegaluk Lagoon were based on reported sightings of whales in the area by Native hunters and field biologists and from aerial surveys [unpublished field notes, 5], much of which is unpublished data [1G, 2G, 3G, 4G].

Multiple factors can influence sighting probability, including observer effort and ocean/sea-ice conditions. Minimum periods of occurrence in the coastal waters of sea-ice region 5 (Cape Lisburne-Icy Cape) were estimated from dates of sightings and hunts off Kasegaluk Lagoon. Considering whale swim speeds and minimum residency times at coastal locations, and the challenges of sighting whales on a given day due to ocean/ice conditions in this region, we estimated minimum periods of occurrence in sea-ice region 5 for single records as 3 days prior to the recorded sighting and 3 days after.

The dates of subsistence harvest of beluga whales in the Mackenzie River delta were used as a proxy of the presence in nearshore waters of beluga whales from the Beaufort Sea stock in the delta. These data were collected as part of an in-depth hunter-based monitoring program of annual subsistence harvest [6]. We recognize that there are limitations to the use of the timing of harvest in estimating period of occurrence. While multiple factors may go into determining the timing of harvest, hunting success is ultimately determined by the presence/absence of whales in the area. We, therefore feel it a reasonable assumption that substantial changes in the dates of arrival or periods of occurrence of beluga whales in the delta will be reflected in the timing of the harvest.

*Sea-ice analysis*

Monthly values of sea ice concentration, SIC, for a given region are a simple average of the fraction of ice in the whole region on the days within that month for which we had data.

To investigate trends in sea-ice over time linear regressions of SIC for each month and region against year and decade were conducted and the slope of the trend line estimated. We used α=0.05 in the statistical test of the null hypothesis, where *p*<0.05 rejected the null hypothesis of no influence of the predictor variable (year, decade) on the response variable (%SIC).

The sea ice data is freely available from the U.S. National Snow and Ice Data Center website, <http://nsidc.org/data/nsidc-0051.html>

**Results and Discussion**

*Genetic analysis*

The results of genetic homogeneity tests of genetic differentiation within mtDNA among beluga whale populations and among sample years within populations are presented in Table SM3 (at the end of this document). Genetic differentiation (*F*_st_) was estimated and statistical significance from 10,000 permutations of the data calculated using Arlequin 3.5.

The Bayesian, model-based clustering method, Structure 2.3.4, was used to infer the population origins of migrating whales from the microsatellite data by determining the likely number of populations, *K*, and assigning individuals to population of origin. As we were interested in inter-annual as well as spatial patterns of migration we included sampling year as well as sampling location as prior information in the analyses. We used a burn-in period of 50,000 iterations followed by 1x10^6^ iterations to collect data. Multiple runs (n=10) were conducted to ensure convergence. Analyses were run both with and without admixture and with and without sampling location/year as prior information to reveal further underlying structure. To identify which individuals were likely immigrants (or descendants of recent immigrants) to their populations, we defined prior probabilities that each individual was an immigrant (*v* = 0.01 - 0.10) and incorporated information on the geographic origin and sampled year of each individual before running the clustering analysis. The results presented in the main text were based on no admixture with location and year priors (figure 2e).

The Structure analysis found that *K*=3 populations was the most likely given the data (Table SM4). As can be seen from figure 2e in the main text the eastern Chukchi Sea (Kasegaluk Lagoon) and the Beaufort Sea (Mackenzie delta) samples formed distinct population clusters. Norton Sound did not, with some whales assigned to the Beaufort Sea population cluster, others assigned to a third cluster and the remainder split between these two. We explore this further in a companion study of population structure in beluga whales across the entire North Pacific where the discrimination of a third genetic cluster in some of the Norton Sound whales becomes more distinct (O’Corry-Crowe et al., unpubl.). As with the eastern Chukchi and Beaufort strata, and in contrast to Norton Sound, the Kotzebue Sound whales from both anomalous years (1996 and 2007) had high likelihoods of coming from the same population; all were clearly assigned to the Beaufort Sea at Q>0.8 (figure 2e).

Table SM4. Likelihood of the number of population clusters, *K*, given the data

| K | Ln *P*(X\|K) | Var [Ln P(D)] | Mean *P*(X\|K) |
| --- | --- | --- | --- |
| 1 | -23824.5 | 40.3 | 23824.4 |
| 2 | -23721.1 | 273.2 | 23721.3 |
| 3 | -23699.0 | 557.4 | 23697.5 |
| 4 | -23706.0 | 627.3 | 23701.2 |

The results of likelihood assignments tests using the program Whichrun 4.1 are summarized in Table SM5. Analyses were conducted on the mtDNA data, the microsatellite data and the two data types combined. The Table summarizes our findings for the combined data set and for the mtDNA data. The likelihood of an individual whale’s haplotype, multi-locus genotype and a combination of the two markers occurring in each of three baseline populations was estimated based on observed haplotype and allele frequencies in each population. The three baseline populations used were the eastern Bering Sea (Norton Sound), eastern Chukchi Sea (Kasegaluk Lagoon) and the Beaufort Sea (Mackenzie delta). The baseline dataset for Norton Sound did not include the test samples from 1996. Assignment confidence of test whales was measured as a log ratio of the haplotype/genotype probabilities of the most likely population of origin compared to the second most likely. We considered an LOD score of ≥0.7 (i.e., a ratio of 5:1) as indicating high confidence in the assigned population being the population of origin.

It should be noted that the sample sizes of the baseline populations differed. This can influence probabilities using this method, especially for rare alleles or haplotypes. In cases where an allele or haplotype in a test individual has not been recorded in one or more baseline populations, Whichrun has the option of seeding that population with that allele/haplotype at a frequency of n=1. This may lead to the adjusted frequency of that variant in those baseline populations with small sample sizes being higher than in others where it was actually observed but with large sample sizes. This was the case with a number of Norton Sound Assignments as it had the smallest sample size (n=73).

Table SM5. Summary of genetic assignment tests of Beluga whales sampled in Kotzebue Sound in 1996 and 2007 and in Norton bay in northern Norton Sound in 1996. Individual whale genetic profiles were tested against nDNA allele and mtDNA haplotype frequencies from three baseline populations. LOD is the log of the ratio of the population probabilities for a whale’s profile in the most likely compared to second most likely population of origin.

|  | Assignments based on highest LOD | | |  | Assignments based on LOD ≥ 0.7 | | |
| --- | --- | --- | --- | --- | --- | --- | --- |
|  | e. Bering Sea | e. Chukchi Sea | Beaufort Sea |  | e. Bering Sea | e. Chukchi Sea | Beaufort Sea |
| mtDNA |  |  |  |  |  |  |  |
| Kotzebue Sound 1996 | 9 | 0 | 4 |  | 9 | 0 | 4 |
| Norton Sound 1996 | 11 | 1 | 10 |  | 0 | 1 | 10 |
| Kotzebue Sound 2007 | 14 | 1 | 25 |  | 0 | 1 | 23 |
|  |  |  |  |  |  |  |  |
| mtDNA - microsatellites |  |  |  |  |  |  |  |
| Kotzebue Sound 1996 | 6 | 1 | 6 |  | 3 | 0 | 3 |
| Norton Sound 1996 | - | - | - |  | - | - | - |
| Kotzebue Sound 2007 | 10 | 3 | 27 |  | 4 | 1 | 22 |

We also investigated the possible role of gender in the atypical genetic profiles observed in Norton and Kotzebue Sounds in 1996 and 2007 as sex segregation has been observed in beluga whales in the wild [7] and gender bias has been recorded in beluga harvests [see 8 for a review]. Some of the gender bias in harvests have been ascribed to hunting preferences, others, however, reflect the sex composition of the herds at time of harvest [8]. In all cases both male and female whales were part of the atypical migration events. Within Kotzebue Sound, we found no evidence for a difference in the sex ratio between samples from the 1996 event and all other non-event years sampled (χ^2^ *p*=0.179). We did, however, find a difference in the case of the 2007 event with a significantly higher proportion of males in that year compared to other non-event years (χ^2^ *p*=0.018). Within Norton Sound, we found no evidence for a difference in the sex ratio between samples from the 1996 event and all other non-event years sampled (χ^2^ *p*=0.182). We did not have the data to assess potential effects or importance of age.

*Beluga sighting data*

Beluga whale sightings near Kasegaluk Lagoon in sea-ice region 5 (Cape Lisburne – Icy Cape) for the 20-year period 1988-2007 are summarized in Table SM6. Minimum periods of occurrence in the coastal waters of sea-ice region 5 were estimated from dates along this coast including hunts off Kasegaluk Lagoon. Considering whale swim speeds, minimum residency times at coastal locations and challenges of sighting whales due to ocean/ice conditions on a given day in this region, minimum periods of occurrence in sea-ice region 5 for single records were estimated as 3 days prior to the recorded sighting and 3 days after (Table SM7).

Table SM6. Beluga whale sightings in coastal waters in the northeast Chukchi Sea between Cape Lisburne and Icy Cape (sea-ice region 35) between 1988 and 2007.

| year | hunt | 2^nd^ hunt | Whales sighted but not hunted | | Whales sighted nearshore | |
| --- | --- | --- | --- | --- | --- | --- |
|  | **date** | **date** | (from shore, boats) | source | (aerial surveys) | source |
| 1988 | 27 Jun |  |  |  |  |  |
| 1989 | 2 Jul |  |  |  |  |  |
| 1990 | 11 Jul |  |  |  | 3 July – and every day in between – 14 Jul | Frost et al. 1993 |
| 1991 | 28 Jun |  |  |  | 4 July – and nearly every day in between – 16 Jul | Frost et al. 1993 |
| 1992 | 6 Jul | 7 Jul |  |  |  |  |
| 1993 | 4 Jul |  |  |  |  |  |
| 1994 | 26 Jun |  |  |  |  |  |
| 1995 | 30 Jun |  |  |  |  |  |
| 1996 | 30 Jun | 4 Jul | 26, 28 and 29 Jun, 5 and 7 Jul | Field notes | 26 Jun – and nearly every day in between – 7 Jul | Lowry et al. 1999 |
| 1997 | 5 Jul |  | 20 Jun, 4 Jul | Field notes |  |  |
| 1998 | 26 Jun |  | 26 Jun | Field notes | 28 Jun, 6 Jul | Lowry et al. 1999 |
| 1999 | 29 Jun |  | 27, 29 and 30 Jun | Field notes |  |  |
| 2000 |  |  |  |  |  |  |
| 2001 | 3 Jul |  |  |  | 4, 5, 6, 7 and 9 Jul | Lowry et al. 2001 |
| 2002 | 7 Jul |  |  |  |  |  |
| 2003 | 28 Jun |  |  |  | 4 and 5 Jul | Lowry et al. 2003 |
| 2004 | 18 Jun |  |  |  |  |  |
| 2005 | 26 Jun |  |  |  |  |  |
| 2006 | 13 Jul |  |  |  |  |  |
| 2007 | 28 Jun | 1 Jul |  |  |  |  |

*Sea-ice analysis*

The springtime sea ice regime of the northern Bering, eastern Chukchi and southern Beaufort Seas are characterized by a northward ice retreat from regions 1-7, but there is considerable variability both inter-annually and between regions (figure SM 1) In line with Arctic-wide trends all regions show a significant negative trend in ice concentration in July (slope = -0.1 to -11.6, *p*<0.05) and there are more negative anomaly years in the latter half of the record. However, it is notable that there is no significant trend in SIC in any region for the month of April (Figure SM1, Table SM8).

In the northern Bering Sea (Region 1 and 2) the greatest variation across years occurred in April-May, with mean ice concentration for May between St. Lawrence Island and Chukotka (Region 1), for example, ranging from 6.8% to 62.7%. In the southeastern Chukchi Sea (Region 3 and 4), the greatest inter-annual variation occurred in late May-early June. In the eastern and northeastern Chukchi Sea (Region 5 and 6), this occurred in June-July, with mean ice concentration between Cape Lisburne and Icy Cape (Region 5), for example, ranging from 2.8% to 91% in June and from 0.5% to 52.3% in July. Finally, the greatest variation in sea ice concentration in the southern Beaufort Sea (Region 7) occurred in July.

Table SM7. Estimates of minimum period of occurrence of beluga whales in coastal waters in the northeast Chukchi Sea between Cape Lisburne and icy Cape.

|  | min. period of occurrence | | |
| --- | --- | --- | --- |
|  | (Region 5: Cape Lisburne - Icy Cape) | | |
| 1988 | 24-Jun | - | 30-Jun |
| 1989 | 29-Jun | - | 5-Jul |
| 1990 | 3-Jul | - | 14-Jul |
| 1991 | 25-Jun | - | 16-Jul |
| 1992 | 3-Jul | - | 9-Jul |
| 1993 | 1-Jul | - | 7-Jul |
| 1994 | 23-Jun | - | 29-Jun |
| 1995 | 27-Jun | - | 3-Jul |
| 1996 | 26-Jun | - | 7-Jul |
| 1997 | 20-Jun | - | 8-Jul |
| 1998 | 23-Jun | - | 6-Jul |
| 1999 | 26-Jun | - | 2-Jul |
| 2000 |  |  |  |
| 2001 | 30-Jun | - | 9-Jul |
| 2002 | 4-Jul | - | 10-Jul |
| 2003 | 25-Jun | - | 5-Jul |
| 2004 | 15-Jun | - | 21-Jun |
| 2005 | 23-Jun | - | 29-Jun |
| 2006 | 10-Jul | - | 16-Jul |
| 2007 | 25-Jun | - | 1-Jul |

To investigate possible links between sea-ice conditions and the occurrence of beluga whales in nearshore waters of the eastern Chukchi Sea and the eastern Beaufort Sea each summer, we compared monthly sea-ice concentrations for all seven regions to the period of occurrence of beluga whales near Kasegaluk Lagoon and the Mackenzie delta, respectively (Table SM9). Sightings of beluga whales and harvest information were used for Kasegaluk Lagoon, while harvest data alone [6] was used for the Mackenzie delta. Spring or summer sea-ice in the northern Bering, eastern Chukchi and southern Beaufort seas did not correlate with period of beluga whale presence in nearshore waters in either area.

Figure SM1. Chart of sea ice concentration (%) for 7 sub-regions in the northern Bering Sea, eastern Chukchi and southern Beaufort Seas over the 36-year time frame: 1979-2014. Positive ice anomaly years are highlighted as blue bars, negative anomaly years by pink bars. Trends in SIC over the 36-year period are indicated by dashed lines with same colors corresponding to the months of April (blue) through July (red).


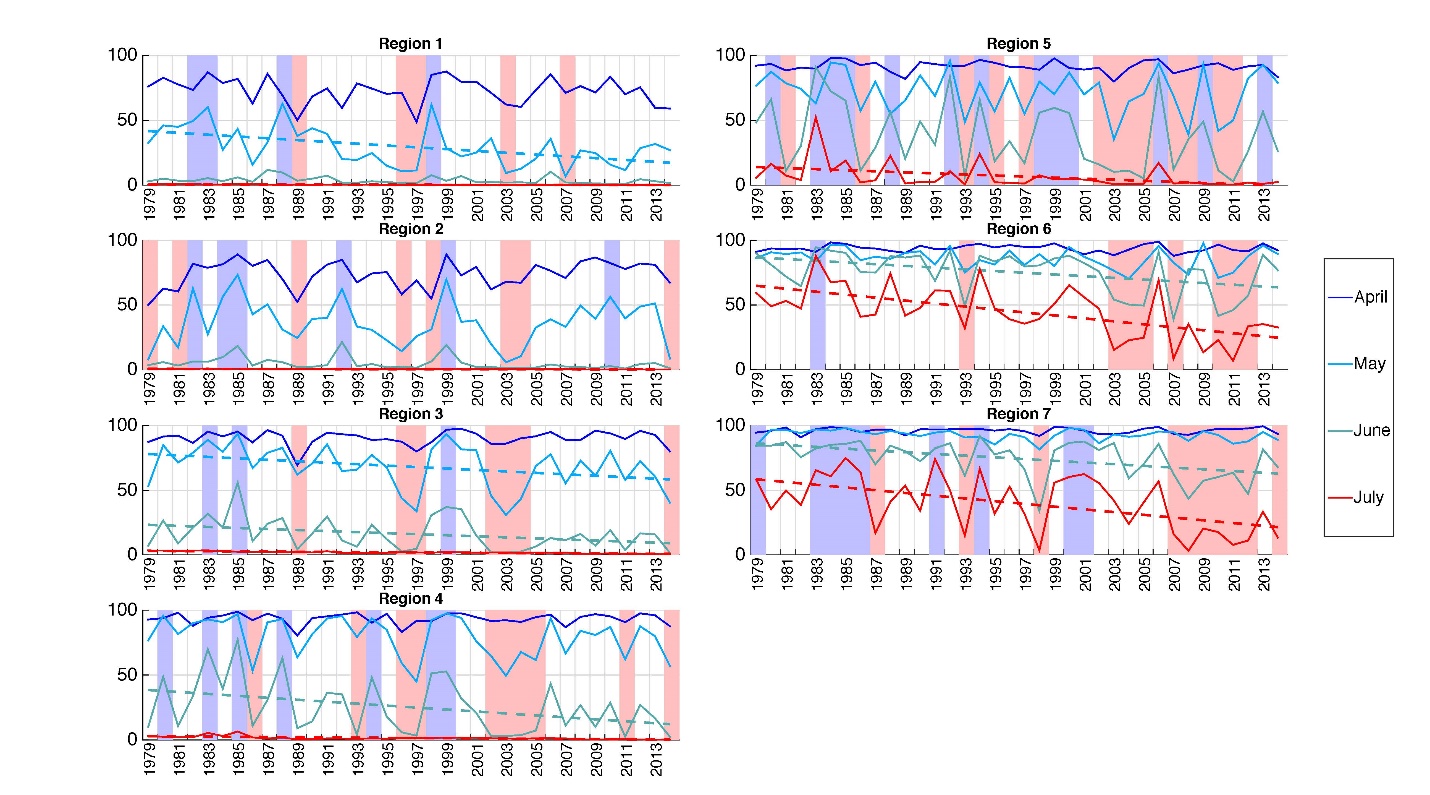


Table SM8. Linear regression values for trends in monthly regional sea ice concentrations for those trends with p-values <0.05. While most of the significant R^2^ values are <0.25, it is notable that slopes are all negative.

| Sea-ice region | Month | R^2^ | *P* value | Slope (%SIC/decade) |
| --- | --- | --- | --- | --- |
| 1 | May | 0.24 | 0.00 | -7.0 |
| 1 | July | 0.53 | 0.00 | -0.1 |
| 2 | July | 0.59 | 0.00 | -0.1 |
| 3 | May | 0.13 | 0.03 | -5.6 |
| 3 | June | 0.12 | 0.04 | -4.1 |
| 3 | July | 0.73 | 0.00 | -0.7 |
| 4 | June | 0.14 | 0.02 | -7.5 |
| 4 | July | 0.38 | 0.00 | -0.8 |
| 5 | July | 0.18 | 0.01 | -4.1 |
| 6 | June | 0.18 | 0.01 | -6.6 |
| 6 | July | 0.37 | 0.00 | -11.5 |
| 7 | June | 0.25 | 0.00 | -6.6 |
| 7 | July | 0.28 | 0.00 | -10.6 |

Table SM9. Correlation (*r*) between beluga whale presence in nearshore waters off Kasegaluk Lagoon and three sub-areas in the Mackenzie delta and spring and summer sea-ice concentration (SIC, %) in seven regions of the northern Bering, eastern Chukchi and southern Beaufort seas.

|  |  |  |  |  |  |  |  |  |
| --- | --- | --- | --- | --- | --- | --- | --- | --- |
|  |  | Region 1 | Region 2 | Region 3 | Region 4 | Region 5 | Region 6 | Region 7 |
|  |  |  |  |  |  |  |  |  |
| Kasegaluk Lagoon | |  |  |  |  |  |  |  |
|  | April | .236 | 0.103 | 0.105 | 0.245 | 0.164 | 0.137 | 0.230 |
|  | May | .255 | 0.333 | 0.217 | 0.249 | 0.433 | 0.616 | 0.153 |
|  | June | .327 | 0.116 | 0.004 | 0.072 | 0.363 | 0.423 | 0.324 |
|  |  |  |  |  |  |  |  |  |
| Mackenzie delta | |  |  |  |  |  |  |  |
| area 1 | April | .191 | 0.315 | 0.216 | -0.003 | 0.363 | 0.326 | 0.372 |
| area 2 | April | -.104 | 0.599 | 0.260 | 0.263 | 0.225 | 0.060 | 0.310 |
| area 3 | April | -.239 | -0.349 | -0.606 | -0.486 | -0.253 | -0.080 | -0.270 |
|  |  |  |  |  |  |  |  |  |
| area 1 | May | -.255 | 0.270 | -0.021 | -0.072 | 0.131 | -0.157 | 0.118 |
| area 2 | May | -.600 | 0.021 | -0.233 | -0.315 | -0.185 | -0.368 | 0.262 |
| area 3 | May | .327 | -0.121 | 0.084 | -0.126 | -0.031 | 0.118 | 0.036 |
|  |  |  |  |  |  |  |  |  |
| area 1 | June | -.357 | 0.361 | -0.031 | -0.075 | 0.012 | 0.089 | 0.262 |
| area 2 | June | -.424 | 0.048 | -0.203 | -0.261 | -0.245 | -0.280 | 0.342 |
| area 3 | June | .109 | -0.217 | -0.036 | -0.040 | -0.030 | 0.257 | 0.113 |
|  |  |  |  |  |  |  |  |  |
| area 1 | July | .144 | 0.363 | 0.303 | 0.302 | -0.254 | -0.065 | 0.339 |
| area 2 | July | -.005 | 0.419 | -0.027 | 0.244 | -0.216 | -0.228 | 0.276 |
| area 3 | July | .284 | -0.058 | 0.294 | -0.368 | -0.031 | 0.061 | 0.228 |
|  |  |  |  |  |  |  |  |  |

*Analysis of Sea ice anomalies and atypical whale migration events*

We used Pearson correlation coefficients (*r*) and chi-square (χ^2^) goodness-of-fit tests to investigate potential links between sea ice anomalies in the Bering, Chukchi and Beaufort Seas and atypical whale migration events recorded in Norton Sound and Kotzebue Sound across the 20-year study (Tables SM10 and 11). As mentioned in the main text, to identify anomalous ice conditions months in which beluga typically occupy each region (April-May for regions 1-3; May-June for regions 4-5; and June-July for regions 6-7) were examined and anomalous occasions were identified when the monthly SIC was 20 percentiles above or below the 36-year mean. To maximize the statistical power, we excluded a number of years from the analyses with small sample size (genetics) or scant sighting data. Thus, our analyses spanned nine years between 1993 and 2002 for Norton Sound, fourteen years between 1994 and 2007 for Kotzebue Sound and 14 years between 1988 and 2007 for Kasegaluk Lagoon. As no anomalous migration or residency patterns were detected in Kasegaluk Lagoon we report findings only for Norton and Kotzebue Sounds in the tables below.

Table SM10. Correlation (*r*) between years with atypical beluga whale migration events in Norton and Kotzebue Sounds and anomalously low and high ice years across seven sea-ice regions in the Bering, Chukchi and Beaufort Seas.

|  | Norton Sound | | Kotzebue Sound | |
| --- | --- | --- | --- | --- |
| Sea Ice Region |  |  |  |  |
|  | Low ice | High Ice | Low Ice | High Ice |
| 1 | 0.66* | -0.13 | 0.65** | -0.11 |
| 2 | 0.66* | -0.13 | 0.19 | -0.11 |
| 3 | 0.50 | -0.13 | 0.12 | -0.11 |
| 4 | 0.40 | -0.25 | 0.06 | -0.17 |
| 5 | -0.32 | -0.25 | 0.06 | -0.26 |
| 6 | -0.13 | - | 0.19 | - |
| 7 | -0.20 | -0.20 | 0.43 | -0.17 |

Significance test of the product-moment correlation: * indicates *p*=0.05, ** indicates *p*<0.01

Table SM11. Goodness-of-fit tests (χ^2^) of observed frequency distributions of typical versus atypical beluga whale migration events to expected frequency distributions based on normal and anomalous ice years across seven sea ice regions in the Bering, Chukchi and Beaufort Seas. The values are *p*-values from a Chi-Square test.

|  | Norton Sound | | Kotzebue Sound | |
| --- | --- | --- | --- | --- |
| Sea Ice Region |  |  |  |  |
|  | Low ice | High Ice | Low Ice | High Ice |
| 1 | 0.047* | 0.708 | 0.016* | 0.672 |
| 2 | 0.047* | 0.708 | 0.469 | 0.672 |
| 3 | 0.134 | 0.708 | 0.649 | 0.672 |
| 4 | 0.236 | 0.453 | 0.825 | 0.533 |
| 5 | 0.343 | 0.453 | 0.825 | 0.334 |
| 6 | 0.708 | - | 0.469 | - |
| 7 | 0.571 | 0.571 | 0.119 | 0.533 |

There were a number of years where we found sea ice anomalies but did not observe atypical migration. It is important to stress that in many of these cases this is because we did not have the samples to make the comparison, and not because we found no correlation. For example, sampling considerations resulted in the exclusion of the years 1989-1992 from our analyses (see figure 2), a period with ice anomalies in a number of regions (figure 1) (1989). Similarly, we have little or no data from Norton Sound for the years 2003-2007 (figure 2), a period with a number of low and high ice years in several regions.

**Literature**

1. O’Corry-Crowe G.M., Suydam R.S., Rosenberg A. *et al*. 1997 Phylogeography, population structure and dispersal patterns of the beluga whale *Delphinapterus leucas* in the western Nearctic revealed by mitochondrial DNA. *Molecular Ecology*, **6**:955-970.
2. O’Corry-Crowe G.M., Dizon A.E., Suydam R.S., and Lowry, L.F. 2002 Molecular genetic studies of population structure and movement patterns in a migratory species: the beluga whale (*Delphinapterus leucas*) in the western Nearctic. *In Molecular and Cell Biology of Marine Mammals*. Ed. C.J. Pfeiffer. Krieger Publishing Co. Malabar, Florida. 53-64.
3. O’Corry-Crowe G.M., Lydersen C., Heide-Jørgensen M.P. *et al*. 2010 Population genetic structure and evolutionary history of North Atlantic beluga whales (*Delphinapterus leucas*) from West Greenland, Svalbard and the White Sea. *Polar Biol.* **33**:1179-1194.
4. O’Corry-Crowe G., Lucey W., Archer F.I., and Mahoney B. 2015 The genetic ecology and population origins of the beluga whales of Yakutat Bay. *Marine Fisheries Review* **71**:47-58.
5. Frost K.J., Lowry L.F., Carroll G. 1993 Beluga whale and spotted seal use of a coastal lagoon system in the northeastern Chukchi Sea. *Arctic* **46**:8-16.
6. Harwood L.A., Kingsley M.C.S., Pokiak F. 2015 Monitoring beluga harvests in the Mackenzie Delta and near Paulatuk, NT, Canada: harvest efficiency and trend, size and sex of landed whales, and reproduction, 1970-2009. *Can. Manuscr. Rep. Fish. Aquat. Sci*. 3059: vi + 32 p.
7. Loseto L.L., Richard P., Stern G.A. *et al*. 2006 Segregation of Beaufort Sea beluga whales during the open-water season. *Can. J. Zool*. **84**:1743-1751.
8. Suydam R.S. 2009. *Age, growth, reproduction and movements of beluga whales (Delphinapterus leucas) from the eastern Chukchi Sea*. Doctoral dissertation. University of Washington, Seattle. 169pp.

**Grey literature**

1. Alaska Beluga Whale Committee beluga whale aerial survey reports: <http://www.north-slope.org/departments/wildlife-management/co-management-organizations/alaska-beluga-whale-committee>.
2. Lowry LF, DeMaster DP, Frost KJ and Perryman W. 1999 Alaska Beluga Whale Committee Surveys of Beluga Whales in the eastern Chukchi Sea, 1996-1998. Alaska Beluga Whale Committee Report 99-01. 20p.
3. Lowry LF and Frost KJ. 2001. Beluga Whale Surveys in the eastern Chukchi Sea, July 2001. Alaska Beluga Whale Committee Report 01-01. 9p.
4. Lowry LF and Frost KJ. 2003. Beluga Whale Surveys in the eastern Chukchi Sea, July 2003. Alaska Beluga Whale Committee Report 03-01. 8p.

Table SM3. Spatial and temporal patterns of genetic differentiation within mtDNA in beluga whales from western Alaska and northwest Canada. Pairwise estimates of F_st_ are below the diagonal. The four colored boxes demarcate the four summering grounds: Norton Sound (eastern Bering Sea stock), Kotzebue Sound, Kasegaluk Lagoon (eastern Chukchi Sea stock) and the Mackenzie Delta (Beaufort Sea stock). Differentiation adjudged to be significant at *p*<0.01 is denoted by dark grey cells, differentiation adjudged to be significant at P<0.05 is denoted by light grey cells.


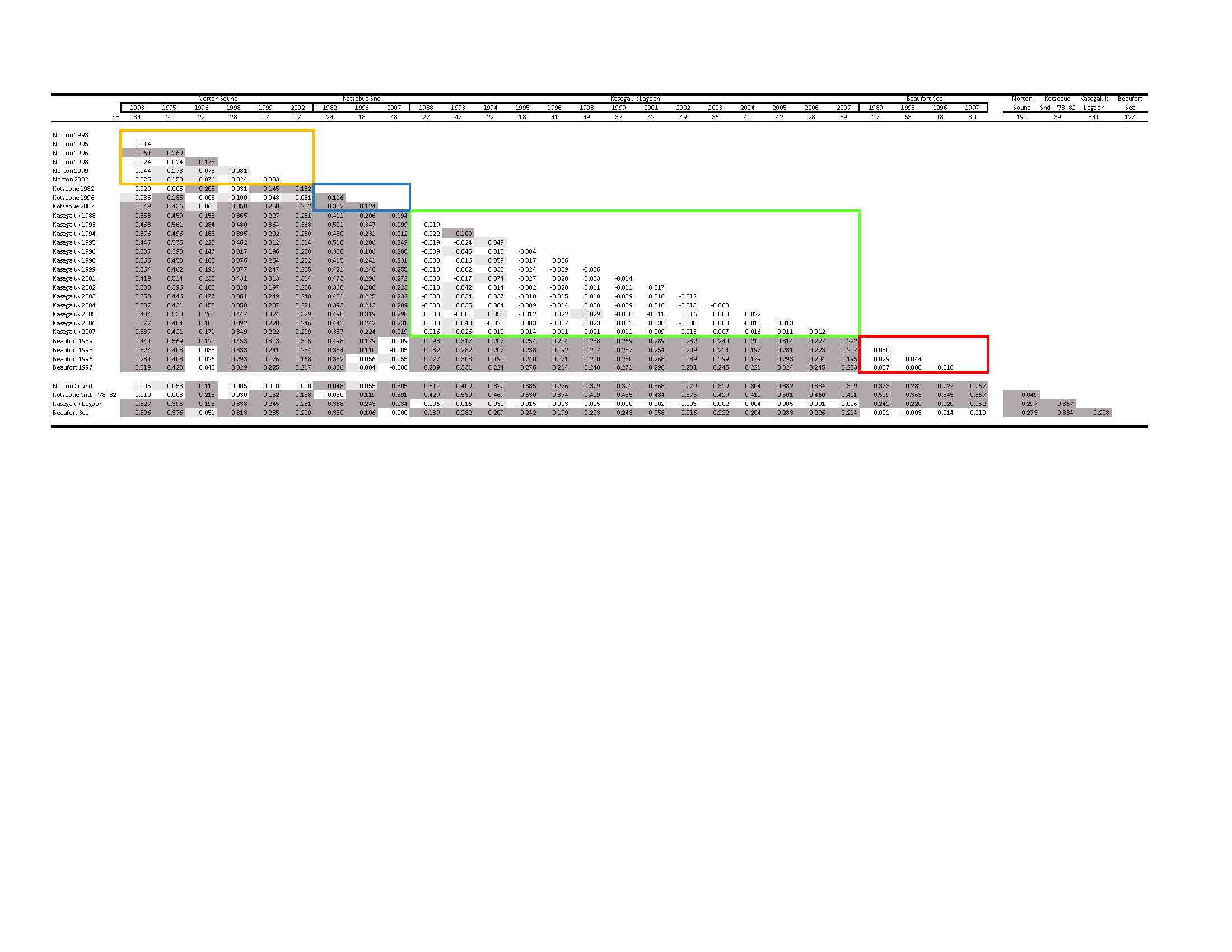

Supplement: O'Corry-Crowe et al - Biology Letters - Supplementary Mat [file rsbl20160404supp1.docx]
